# Probiotics as a Complementary Therapy for Management of Obesity: A Systematic Review

**DOI:** 10.1155/2021/6688450

**Published:** 2021-01-22

**Authors:** Salman Shirvani-Rad, Ozra Tabatabaei-Malazy, Shahrzad Mohseni, Shirin Hasani-Ranjbar, Ahmad-Reza Soroush, Zahra Hoseini-Tavassol, Hanieh-Sadat Ejtahed, Bagher Larijani

**Affiliations:** ^1^Obesity and Eating Habits Research Center, Endocrinology and Metabolism Clinical Sciences Institute, Tehran University of Medical Sciences, Tehran, Iran; ^2^Non-Communicable Diseases Research Center, Endocrinology and Metabolism Population Sciences Institute, Tehran University of Medical Sciences, Tehran, Iran; ^3^Endocrinology and Metabolism Research Center, Endocrinology and Metabolism Clinical Sciences Institute, Tehran University of Medical Sciences, Tehran, Iran

## Abstract

**Background:**

Considering the observed role of probiotics in modulating gut microbiome, probiotics are discussed to be one potential complementary therapy for obesity management in recent years. The aim of the present study was to systematically review the meta-analyses of controlled trials and investigate the effects of probiotics on obesity.

**Methods:**

A comprehensive search was conducted on PubMed, Web of Science, and Cochrane Library web databases up to May 2020. Inclusion criteria were meta-analyses of controlled trials which evaluated the impact of probiotics on obesity in English language. Meta-analyses done on pregnant women, children, animal studies, or the effect of prebiotics on anthropometric indices were excluded.

**Results:**

Within 325 recorded studies, 20 studies met the inclusion criteria consisting of 16676 overweight/obese adults with different underlying disorders such as nonalcoholic fatty liver disease (NAFLD), or polycystic ovary syndrome (PCOS). The length of intervention varied from 2 to 26 weeks. Results of meta-analyses have shown a moderate effect of probiotics on body weight in overweight/obese adults: from −0.526 kg/m^2^ (95% CI: −0.810, −0.247) to −0.25 kg/m^2^ (95% CI: −0.33, −0.17). Body mass index (BMI) was changed from −1.46 kg/m^2^ (95% CI: −2.44, −0.48) to −1.08 kg/m^2^ (95% CI: −2.05, −0.11) in NAFLD. Probiotics could reduce BMI from −0.36 kg/m^2^ (95% CI: −0.74, 0.02) to −0.29 kg/m^2^ (95% CI: −0.54, −0.03) in patients with PCOS.

**Conclusion:**

It seems that the probiotic products could have beneficial effects as an adjunct therapy for care and management of obesity when used in high dose. However, due to heterogeneity of included studies, it is required to confirm our results by more meta-analyses of clinical trials.

## 1. Introduction

During recent decades, prevalence of overweight and obesity, which are a consequence of more energy receiving and less energy consumption, considerably increased among different age groups [1–3]. Obesity is explained as a body mass index (BMI) of 30 kg/m^2^ or more while overweight is defined as a BMI between 25 and 30 kg/m^2^ [4]. According to the World Health Organization (WHO), 1.9 billion individuals aged ≥18 years old were overweight (39%) worldwide, 650 million of which (13%) were obese in 2016. Considering that prevalence rate of obesity increased to three times since 1975 [4], obesity is changing to a major global health concern with high burden on healthcare systems [5–7]. Both genetic and environmental factors such as sedentary lifestyle, urbanization, and easy access to high-energy foods are considered as reasons for the rapid rise in the prevalence rate of obesity and overweight [5, 8–10]. Changing lifestyle and personal behaviors are believed as best ways of treating obesity for a long time [3]. New studies which found evidences for concurrent dysbiosis of microbiota and obesity prevalence suggested the possible association between obesity and microbiome [1, 2, 11]. It means the gut microbiome is involved in regulation of energy intake and expenditure.

The underlying mechanisms by which the gut microbiota can contribute to the weight management include many pathways; for instance, saccharolytic gut microbes are able to generate short-chain fatty acids (SCFAs) by fermentation of indigestible polysaccharides. The gut microbiota-derived SCFAs have critical roles in decreasing oxidative stress and inflammation as well as regulating energy consumption. Moreover, SCFAs are responsible for enhancement of satiation through reducing gut motility and stimulating secretion of glucagon-like peptide 1 (GLP1) and peptide YY (PYY) [2, 8, 12].

So, microbial intervention like probiotic foods and supplements could be a novel approach for controlling obesity [11–13]. Probiotics are defined as live microorganisms that are supposed to have positive effects when consumed in acceptable and enough quantities [3, 6]. Using probiotics is suggested as a potential new approach but still doubted for reforming dysbiosis of gut microbiota composition to control obesity through improvement of BMI, body weight (BW), waist circumference (WC), or body fat mass (BFM) [2, 6, 12]. Results of previous studies regarding impact of probiotics on obesity showed contradictions; for instance, some studies did not show any obvious change in anthropometric indices [6, 7, 14, 15]; in contrast, some other studies reported significant changes [1–3, 5, 8, 11–13, 16–22]. These paradoxical and varying results were a motivation for us to look over related studies to answer accurately whether probiotics have beneficial effects on obesity or not. Therefore, the aim of this systematic review study is to gather and review results of meta-analysis studies investigating the effect of probiotics consumption on obesity.

## 2. Methods

### 2.1. Search Strategy

PubMed, Web of Science, and Cochrane Library web databases were comprehensively searched for meta-analysis studies evaluating impact of probiotics on obesity recorded up to May 2020. The search terms were “probiotic,” “probiotics,” “*Lactobacillus*,” “*Bifidobacterium*,” “obese,” “overweight,” “obesity,” “body weight,” “adiposity,” “fat mass,” “weight,” “BMI,” “waist circumference,” “body mass index,” and “meta-analysis”. All of the articles were inspected and duplicate ones were removed manually operated by two independent researchers.

#### 2.1.1. Eligibility Criteria and Study Selection

At first, all documents were checked over for titles and abstracts by two researchers independently followed by reviewing the full-text articles based on inclusion and exclusion criteria. The inclusion criterion was meta-analyses performed on controlled trials investigating the impact of various probiotic products on anthropometric indices. The probiotic products consisted of probiotic foods or probiotic supplements. Moreover, probiotic foods are defined as fermented foods that naturally contain probiotics, or have probiotics added to them. The exclusion criteria were (1) meta-analyses that were done on pregnant women, children, or animals and (2) meta-analyses on the effect of prebiotics on anthropometric indices. English language was considered as search limitation.

Preferred Reporting Items for Systematic reviews and Meta-Analyses (PRISMA) guideline was used for performing this study [23].

### 2.2. Data Extraction

Data were extracted from the articles including authors and publication year, number and type of included studies in the meta-analysis, participants' characteristics (sample size, age, sex, and underlying disorder), type and dose of intervention, duration of interventions, main outcomes, and reported side effects.

### 2.3. Quality Assessment

The critical appraisal tool entitled Assessment of Multiple Systematic Reviews (AMSTAR) was used for quality assessment of the included studies [24]. The quality of studies was defined as high quality for scores 8–11, average quality for scores 4–7, and poor quality for scores ≤3. All of the mentioned steps including search, study selection, data extraction, and quality assessment were done by two researchers independently and any disagreement was resolved by discussing until reaching a consensus.

## 3. Results and Discussion

### 3.1. Results

**Figure 1:**
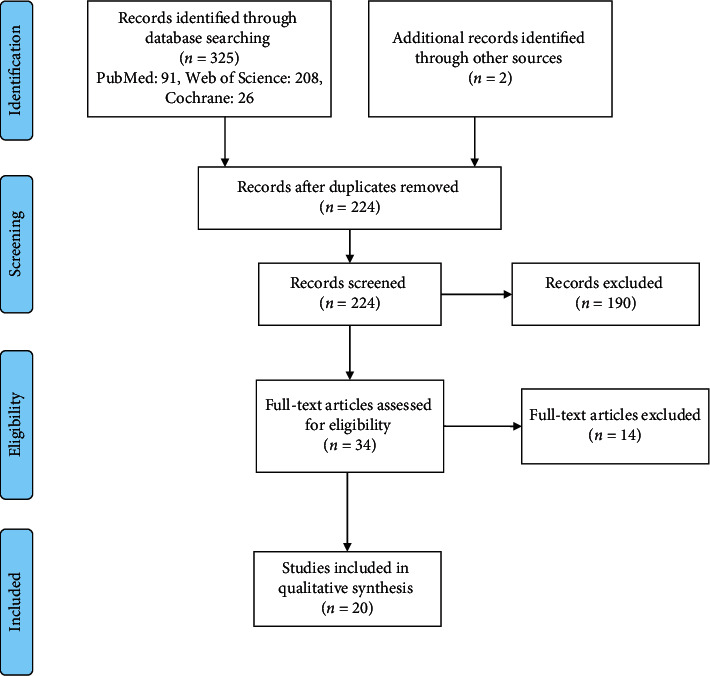
Flow diagram of the study selection process.

After removing duplicate articles (101) from primary recorded studies (325), 224 articles remained to assess their title/abstract and full text. Finally, 20 articles were included in this systematic review (Figure 1). Characteristics of the included meta-analyses are presented in Table 1. All included studies were meta-analysis of randomized controlled trials (RCTs) conducted on both genders of adults for 2–26 weeks [1–3, 5–8, 11–22, 25]. Total sample size was 16676 overweight/obese subjects with different underlying disorders such as nonalcoholic fatty liver disease (NAFLD) or polycystic ovary syndrome (PCOS). Various formats of probiotic products such as fermented foods, fermented dairy products, or supplements including capsules, powder, and sachets which contained single or multiple diverse species were utilized.

Totally, in 12 articles carried on obese or overweight adults from both genders, participants were treated with probiotic capsules, probiotic powder, yogurt, fermented milk, or dough containing various species such as *Lactobacillus, Propionibacterium, Bifidobacterium*, and *Acetobacter* [1–3, 5–8, 11–13, 17, 22]. In most of the studies [1–3, 5, 8, 11–13, 17, 22], significant changes in anthropometric indices were shown. The greatest and the least observed changes in BMI were −0.526 kg/m^2^ (95% CI: −0.810, −0.247) and −0.25 kg/m^2^ (95% CI: −0.33, −0.17) through 3–12 weeks of intervention, respectively [1, 22]. BW was another index which showed significant reduction through probiotic supplementation; regarding that, the greatest reduction was −0.65 kg (95% CI: −1.12, −0.18) and the least reduction was −0.26 kg (95% CI: −0.43, −0.09) during 4–24 weeks [8, 17]. Moreover, other anthropometric indices, including BFM and WC, were reduced in a range from −0.94 kg (95% CI: −1.17, −0.72) to −0.30 kg (95% CI: −0.48, −0.12) [8, 17] and from −2.11 cm (95%CI: −3.543, −0.677) to −0.35 cm (95% CI: −0.81, 0.11), respectively [13, 22].

Six studies out of 20 total included studies were accomplished on both genders of obese adult patients with nonalcoholic fatty liver disease using diverse probiotic products as intervention constituted by various species like *Streptococcus cerevisiae*, *Streptococcus thermophilus*, and *Lactobacillus* spp. [14, 15, 18, 19, 21, 25]. Three of these studies showed no significant change in anthropometric indices [14, 15, 25], contrasting to others [18, 19, 21]. Among the studies displaying beneficial effects on BMI, two of them showed better results when *Bifidobacterium* and *Lactobacillus* species were administered (−1.46 kg/m^2^ (95% CI: −2.44, −0.48) and −1.08 kg/m^2^ (95% CI: −2.05, −0.11)) [18, 19].

Two meta-analyses among 20 included studies (16, 20) reported effects of 8–12 weeks intervention by probiotic on adult obese women with PCOS. In the study by Hadi et al. [16], probiotic supplementation was observed to be more effective in reducing BW for participants >30 years old but in contrast no special difference was seen in subgroup analyses for age in Tabrizi et al.'s study [20].

Hadi et al. [16] and Tabrizi et al. [20] assessed the effect of probiotics contained in capsules. In both studies, supplements contained different species of *Lactobacillus* and *Bifidobacterium* genera such as *L. acidophilus, L. casei, L. rhamnosus, L. reuteri,* and *B. bifidum.* Both meta-analyses reported change in BMI and BW. BMI was reduced from −0.36 kg/m^2^ (95% CI: −0.74, 0.02) to −0.29 kg/m^2^ (95% CI: −0.54, −0.03) and also BW showed reduction varying from −1.3 kg (95% CI: −1.93, −0.13) to −0.30 kg (95% CI: −0.53, −0.07) [16, 20].

The effects of probiotics dosage and single/multi-strains were assessed in subgroup analyses of Wang et al.'s [2], John et al.'s [8], and Koutnikova et al.'s [5] studies. Wang et al. found a significant reduction in BW with high dose of probiotics (−0.58 kg, 95% CI: −0.92, −0.23), and a single strain of probiotics (−0.49 kg, 95% CI: −0.92, −0.07). Similar effects were reported for BMI when the high dose was used (−0.29 kg/m^2^, 95% CI: −0.46, −0.12) and single strain of probiotics (−0.36 kg/m^2^, 95% CI: −0.52, −0.20) [2]. In John et al.'s [8] study, a greater significant reduction in BMI was shown with high dose of probiotics (−0.43 kg/m^2^, 95% CI: −0.56, −0.30) compared to low dose of probiotics. When stratified by single vs. multi-strain probiotic supplementation, a significant decrease in BMI (−0.41 kg/m^2^, 95% CI: −0.56, −0.27), BW (−0.77 kg, 95% CI: −1.52, −0.03), and fat mass (−0.95 kg, 95% CI: −1.19, −0.71) was observed by single strain. In subgroup analysis in Koutnikova et al.'s [5] study, a significant effect on BW and BMI when three or more bacterial species were used was observed.

No serious side effect has been reported in the included studies. Just in Million et al.'s study [7], weight gain has been reported after supplementation with some species of *L. acidophilus*, *L. delbrueckii*, and *L. plantarum*. Regarding the quality of included meta-analyses studies, most of the studies had AMSTAR score ≥8 (90%, 18 of 20 articles) which was interpreted as high quality [1–3, 5–8, 11–15, 17–20, 22, 25] and two other studies were classified as medium quality [16, 21].

### 3.2. Discussion

This systematic review of meta-analyses was aimed at uncovering the effects of probiotics usage on obesity/overweight indices including BW, BMI, BFM, and WC. Results varied from a no significant change to a significant decrease during 2–26 weeks' interventions. Regarding the observed improvement of anthropometric indices, from 20 meta-analyses included in the current study, 10 studies disclosed significant change in BW [2, 3, 5, 8, 11, 12, 16, 17, 19, 20], 14 studies showed significant change in BMI [1–3, 5, 8, 11, 12, 16–22], 6 studies reported change in WC [1, 2, 5, 13, 17, 22], and 4 studies disclosed change in BFM [2, 5, 8, 17]. In the study by Koutinkova et al., an improvement in anthropometric indices was reported when ≥3 species were used [5]. In contrast, John et al. [8] and Wang et al. [2] represented that probiotics containing only single bacterial strain had a considerable decreasing effect on BMI and BW. Moreover, in John et al.'s study [8], it was implicated that even lower dose of interventions can induce a considerable reduction in BMI and BW when interventions continued for more than 12 weeks [8].

Obesity as one of the main health problems predisposes people to cardio-metabolic disorders like type 2 diabetes mellitus and cardiovascular diseases [3]. Probiotic products can affect obesity via some proposed underlying mechanisms [3]. Modulation of gut microbiota composition and function has been suggested as one of these mechanisms. Dysbiosis is common in most obese individuals which can be reformed through probiotic consumption [16]. Moreover, probiotics are able to ameliorate inflammation by inducing secretion of SCFAs and concurrently decreasing number of bacteria producing lipopolysaccharides. SCFAs may lead to regulating energy hemostasis via stimulation of enterocyte receptors and secretion of glucagon-like peptide 1 (GLP1) and peptide YY (PYY) [1, 2, 16]. Furthermore, probiotics stimulate release of glucagon-like peptide 2 (GLP2) which leads to higher expression of tight junction proteins, better gut barrier function, and ultimately more effective inflammation control [26]. Following suppression of inflammation, insulin resistance is also improved in obese individuals [20]. In a meta-analysis study by Companys et al. [17], probiotic supplements together with dairy products generally showed reduction in different anthropometric indices. However, when *L. acidophilus, L. gasseri* SBT2055, and *B. lactis* BB12 have been added to the combination, with intervention for more than 12 weeks at the dosage level of 10^7^ to 10^11^ (CFU/day), a bigger reduction in BW, BFM, and BMI was observed. These reductions were even reported in greater amount when participants had low calorie diet synergistically. Zhang et al. reported the greater effects for probiotics supplementation with duration of ≥8 weeks which contained more than 1 species regardless of type of species [3]. In the study of Park S et al., no change in anthropometric parameters was observed via any kind of probiotics therapy but exceptionally when fermented milk including *Lactobacillus* spp. with dosage of 10^8^ to 10^9^ CFU was utilized, it brought significant changes afterwards [6]. There are controversies in results of meta-analyses investigating probiotics impact on anthropometric indices. Most of meta-analyses showed significant desired changes in BW, BMI, and WC [2, 3, 5, 8, 11–13, 17, 22] and some others did not report any change [6, 7]. These controversies could have originated from differences in duration of interventions, the probiotic dosage, or characteristics of participants. Moreover, the probiotic carriers can play an important role in their efficacy and the effect of probiotics is species- and strain-specific [26]. Since most of the studies used a mixture of different probiotic species, we cannot identify the species with the most considerable effects. The observed effects in studies may be caused by synergistic function of different species used together in probiotics. So, further studies are needed to determine the most effective strains of probiotics with anti-obesity properties.

The safety of probiotic products is a matter of concern especially for infants, people suffering from cancer, critically ill, and immunocompromised patients [27]. The principal reported adverse effects ranged from mild such as gastrointestinal side effects and skin complications to severe including inflammation of endocardium and systemic infections [27]. Therefore, because of mostly unknown probable deleterious effects, it is better to do more research before general recommendation of probiotics [28].

This study has some strengths and limitations. The main strength of this systematic review was comprehensive evaluation of the meta-analyses of high qualified RCTs concerning the anti-obesity effect of probiotic products. Somehow, as a major limitation of the present study, the use of different mixture of probiotic strains made it difficult to draw exact conclusion about each probiotic strain.

## 4. Conclusion

In conclusion, it seems that using various high-dose probiotic products or supplements could improve overweight/obese indices in participants with different underlying disorders. However, since these products may not be without side effects for all groups, the risk-benefit assessment should be done prior to their prescription.

## Figures and Tables

**Table 1:** Included meta-analysis studies investigating the effect of probiotics on obesity. Probiotic foods consisted of fermented foods that naturally contain probiotics or foods which have probiotics added to them.

Authors/published year	Included studies in meta-analysis (*n*)	Type of included studies in meta-analysis	Total sample size (*n*)	Participants' characteristics	Type and dose of intervention	Duration of interventions (weeks)	Main outcomes	Risk of bias assessment	Reported side effects	AMSTAR score
Hadi et al., 2020	3 (all in Iran)	RCTs	180 (intervention: UN, control: UN)	Women with PCOS ≥18 y	Different species of probiotic supplements (10^9^ CFU/day)	12	−Sig. ↓weight: −1.3 kg (95% CI: −1.93, −0.13)	Yes	No side effects	7
							−Sig. ↓BMI; −0.36 kg/m^2^ (95% CI: −0.74, 0.02)			

Company et al., 2020	17 (11 in Asian countries, 6 in European countries, 1 in Brazil)	DBRCTs	1,106 (intervention: 486, placebo: 62)	Cardio-metabolic disease subjects with obesity, adults ≥18 y, both sexes	Multiple and single species of probiotic supplements within powder and capsule (10^8^–10^11^ CFU/day)	4–12	−sig. ↓BW: −0.26 kg (95% CI: −0.43, −0.09)	Yes	No side effects	11
							−sig. ↓BMI: −0.35 kg/m^2^ (95% CI: −0.48, −0.22)			
							−sig. ↓WC: −0.37 cm (95% CI: −0.52, −0.21)			
							−sig. ↓BFM: −0.30 kg (95% CI: −0.48, −0.12)			

Cao et al., 2020	31 (22 in Asian countries, 6 in European countries, 3 in Brazil)	RCTs	2051 (intervention: UN, control: UN)	Obese and overweight adults ≥18 y, both sexes	Probiotic foods and supplements and dairy product (10^8^–10^11^ CFU/day)	3–12	−sig. ↓BMI: −0.25 kg/m^2^ (95% CI: −0.33, −0.17)	Yes	No side effects	9
							−sig. ↓WC: −0.99 cm (95% CI: −1.33, −0.66)			

Xiao M et al., 2019	12 (9 in Asian countries, 3 in European countries)	DBRCTs	693 (intervention: UN, control: UN)	Adults with nonalcoholic fatty liver disease, both sexes	Different species of probiotic supplements (UN amounts)	8–24	−sig. ↓BMI: −1.46 kg/m^2^ (95% CI: −2.44, −0.48)	Yes	No serious side effects	10

Wang et al., 2019	12 (9 in Asian countries, 2 in European countries, 1 in Brazil)	DBRCTs, SBRCTs	821 (intervention: 405, control: 416)	Adults with BMI ≥25 and ≥18 y, both sexes	Multiple and single species of probiotic supplements (10^7^–10^11^ CFU/day)	8–26	−sig. ↓BW: −0.55 kg (95% CI: −0.91, −0.19)	Yes	No side effects	9
							−sig. ↓BMI: − 0.30 kg/m^2^ (95% CI: −0.43, −0.18)			
							−sig. ↓WC: −1.20 cm (95%CI: −2.21, −0.19)			
							−sig. ↓BFM: −0.91 kg (95% CI: −1.19, −0.63)			

Tang et al., 2019	12 (7 in Asian countries, 4 in European countries, 1 in Brazil)	DBRCTs	805 (intervention: 407, control 398)	Adults with nonalcoholic fatty liver disease, both sexes	Multiple and single species of probiotic supplements (varied amounts)	8–24	−sig. ↓BW: −2.31 kg (95% CI: −4.45, −0.16) sig. ↓BMI: −1.08 kg/m^2^ (95% CI: −2.05, −0.11)	Yes	No side effects	10

Tabrizi et al., 2019	7 (all in Iran)	RCTs	415 (intervention: 212, control: 213)	Women with PCOS ≥18 y	Probiotic capsule supplements (10^8^–10^10^ CFU/day)	8–12	−sig. ↓BW: −0.30 kg (95% CI: −0.53, −0.07)	Yes	No side effects	9
							−sig. ↓BMI: −0.29 kg/m^2^ (95% CI: −0.54, −0.03)			

Liu et al., 2019	4 (2 in Iran, 2 in European countries)	RCTs	218 (intervention: 110, control: 108)	Adults with nonalcoholic fatty liver disease ≥18 y, both sexes	Different species of probiotic supplement (varied amounts)	8–12	No significant effect on BMI or WC	Yes	Rare and mild reverse reactions	9

Koutinkova et al., 2019	68 (26 in Iran)	RCTs, COTs	4015 (intervention: UN, control: UN)	Overweight/obese/normal weight adults, both sexes	Multiple and single species of probiotic products (varied amounts)	2–28	−sig. ↓BW: −0.39 kg (95% CI: −0.57, −0.21)	Yes	No side effects	11
							−sig. BMI: −0.33 kg/m^2^ (95% CI: −0.53, −0.12)			
							−sig. WC: −1.01 cm (95% CI: −1.55, −0.48)			
							−sig. BFM: −0.62 kg (95% CI: −0.91, −0.34)			

Dong et al., 2019	6 (UN countries)	DBRCTs	476 (intervention: UN, control: UN)	Adults with BMI ≥25 and ≥18 y, both sexes	Probiotic capsules and probiotic dairy products (10^6^–10^11^ CFU/day)	8–24	No significant effect on BMI or BFM	Yes	No side effects	11
							−sig. ↓WC: −0.35 cm (95% CI: −0.81, 0.11)			

John et al., 2018	18 (15 in Asian countries, 1 in Finland, 1 in Brazil, 1 in Canada)	RCTs	803 (intervention: 443, control: 360	Adults ≥18 y with BMI ≥25, both sexes	Multiple and single species of probiotic food or supplements (10^7^–10^11^ CFU/day)	4–24	−sig. ↓BW: −0.65 kg (95% CI: −1.12, −0.18)	Yes	No side effects	10
							−sig. ↓BMI: −0.33 kg/m^2^ (95% CI: −0.47, −0.18)			
							−sig. ↓BFM: −0.94 kg (95% CI: −1.17, −0.72)			

Borgeraas et al., 2018	13 (10 in Asian countries, 1 in European countries, 2 in the US)	DBRCTs, SBRCTs	737 (intervention: 369, control: 368)	Overweight or obese adults ≥18 y	Probiotic dairy products and supplements (10^9^–10^11^ CFU/day)	3–12	−sig. ↓BW: −0.6 kg (95% CI: −1.19, −0.01)	Yes	No side effects	11
							−sig. ↓BMI: −0.27 kg/m^2^ (95% CI: −0.45, −0.08)			

Dror et al., 2017	15 (7 in Asian countries, 4 in European countries, 2 in Canada, 2 in the US)	RCTs	921 (intervention: 468, control: 453)	Normal or obese or overweight adults ≥18 y	Different species of probiotic dairy and supplements (10^7^–10^8^ CFU/day)	8–12	−sig. ↓BW: −0.43 kg (95% CI: −0.67, −0.2)	Yes	No side effects	9
							−sig. ↓BMI: −0.43 kg/m^2^ (95% CI: −0.54, −0.33)			

Lavekar et al., 2017	3 (2 in European countries, 1 in Asian countries)	DBRCTs	157 (intervention: 79, control: 78)	Adults with nonalcoholic fatty liver disease	Multiple and single probiotic species (varied amounts)	12–24	−sig. ↓BMI: −1.45 kg/m^2^ (95% CI: −3.06, 0.16)	Yes	No serious side effects	6

Zhang et al., 2016	25 (UN countries)	DBRCTs, RCTs	1931 (intervention: 977, control: 954)	Adults ≥18 y, both sexes	Multiple and single species of probiotic supplements (varied amounts)	3–24	−sig. ↓BW: −0.59 kg (95% CI: −0.87, −0.30)	Yes	No side effects	10
							−sig. ↓BMI: −0.49 kg/m^2^ (95% CI: −0.74, −0.24)			

Gao et al., 2016	6 (4 in Asian countries, 2 in European countries)	RCTs	205 (intervention: 115, control: 90)	Adults with nonalcoholic fatty liver disease, both sexes	Multiple and single species of probiotic sources (NA)	12–24	No significant effect on BMI	Yes	Few side effects	10

Sun et al., 2015	5 (1 in Korea, 1 in Japan, 1 in Iran)	DBRCTs	NA	Obese adults ≥18 y, both sexes	Different species of probiotic capsules and probiotic fermented milk (10^7^–10^10^ CFU/day)	8–12	−sig. ↓BMI: −0.526 kg/m^2^ (95% CI: −0.810, −0.247)	Yes	No side effects	11
							−sig ↓WC: −2.11 cm (95% CI: −3.543, −0.677)			

Park et al., 2015	9 (NA)	RCTs	689 (intervention: UN, control: UN)	Obese and overweight adults ≥18 y, both sexes	Probiotic supplements and dairy products (10^6^–10^10^ CFU/day)	3–24	No significant effect on BW or BMI	Yes	No serious side effects	9

Ma et al., 2013	4 (1 in Hong Kong, 1 in Spain, 1 in Italy, 1 in the US)	DBRCTs	134 (intervention: 68, control: 66)	Nonalcoholic patients, both sexes	Multiple and single species of probiotic sources (NA amounts)	8–24	No significant effect on BMI	Yes	No side effects	8

Million et al., 2012	5 (UN countries)	DBRCTs	319 (intervention: 257, control: 162)	Lean and obese adults ≥18 y, both sexes	Multiple and single species of probiotic sources (10^7^–10^10^ CFU/day)	4–24	No significant effect on BW	Yes	Weight gain in usage of *L. acidophilus, L. delbrueckii, L. plantarum*	10

BMI: body mass index; WC: waist circumference; BFM: body fat mass; BW: body weight; RCTs: randomized controlled trials; DBRCTs: double blind randomized controlled trials; SBRCTs: single blind randomized controlled trials; COTs: cohort study; PCOS: polycystic ovary syndrome; CFU: colony forming units; UN: unknown; NA: not available; CI: confidence interval.

## Data Availability

All data analyzed in this work are supported by the published articles in PubMed, Web of Science, and Cochrane Library web databases, and all data generated are included in this published article.

## References

[1] Cao S., Ryan P. M., Salehisahlabadi A. (2020). Effect of probiotic and synbiotic formulations on anthropometrics and adiponectin in overweight and obese participants: a systematic review and meta-analysis of randomized controlled trials. *Journal of King Saud University-Science*.

[2] Wang Z. B., Xin S. S., Ding L. N. (2019). The potential role of probiotics in controlling overweight/obesity and associated metabolic parameters in adults: a systematic review and meta-analysis. *Evidence-Based Complementary and Alternative Medicine*.

[3] Zhang Q., Wu Y., Fei X. (2016). Effect of probiotics on body weight and body-mass index: a systematic review and meta-analysis of randomized, controlled trials. *International Journal of Food Sciences and Nutrition*.

[4] Obesity and Overweight, 2020, https://www.who.int/en/news-room/fact-sheets/detail/obesity-and-overweight

[5] Koutnikova H., Genser B., Monteiro-Sepulveda M. (2019). Impact of bacterial probiotics on obesity, diabetes and non-alcoholic fatty liver disease related variables: a systematic review and meta-analysis of randomised controlled trials. *Bmj Open*.

[6] Park S., Bae J.-H. (2015). Probiotics for weight loss: a systematic review and meta-analysis. *Nutrition Research*.

[7] Million M., Angelakis E., Paul M., Armougom F., Leibovici L., Raoult D. (2012). Comparative meta-analysis of the effect of Lactobacillus species on weight gain in humans and animals. *Microbial Pathogenesis*.

[8] John G. K., Wang L., Nanavati J., Twose C., Singh R., Mullin G. (2018). Dietary alteration of the gut microbiome and its impact on weight and fat mass: a systematic review and meta-analysis. *Genes*.

[9] Le Chatelier E., Nielsen T., Qin J. (2013). Richness of human gut microbiome correlates with metabolic markers. *Nature*.

[10] Reilly J. J., Armstrong J., Dorosty A. R. (2005). Early life risk factors for obesity in childhood: cohort study. *Bmj*.

[11] Dror T., Dickstein Y., Dubourg G., Paul M. (2017). Microbiota manipulation for weight change. *Microbial Pathogenesis*.

[12] Borgeraas H., Johnson L. K., Skattebu J., Hertel J. K., Hjelmesaeth J. (2018). Effects of probiotics on body weight, body mass index, fat mass and fat percentage in subjects with overweight or obesity: a systematic review and meta-analysis of randomized controlled trials. *Obesity Reviews*.

[13] Dong Y., Xu M., Chen L., Bhochhibhoya A. (2019). Probiotic foods and supplements interventions for metabolic syndromes: a systematic review and meta-analysis of recent clinical trials. *Annals of Nutrition and Metabolism*.

[14] Liu L., Li P., Liu Y., Zhang Y. (2019). Efficacy of probiotics and synbiotics in patients with nonalcoholic fatty liver disease: a meta-analysis. *Digestive Diseases and Sciences*.

[15] Ma Y.-Y. (2013). Effects of probiotics on nonalcoholic fatty liver disease: a meta-analysis. *World Journal of Gastroenterology*.

[16] Hadi A., Moradi S., Ghavami A., Khalesi S., Kafeshani M. (2020). Effect of probiotics and synbiotics on selected anthropometric and biochemical measures in women with polycystic ovary syndrome: a systematic review and meta-analysis. *European Journal of Clinical Nutrition*.

[17] Companys J., Pla-Pagà L., Calderón-Pérez L. (2020). Fermented dairy products, probiotic supplementation, and cardiometabolic diseases: a systematic review and meta-analysis. *Advances in Nutrition*.

[18] Xiao M. W., Lin S.-X., Shen Z.-H., Luo W.-W., Wang X.-Y. (2019). Systematic review with meta-analysis: the effects of probiotics in nonalcoholic fatty liver disease. *Gastroenterology Research and Practice*.

[19] Tang Y., Huang J., Zhang W. Y. (2019). Effects of probiotics on nonalcoholic fatty liver disease: a systematic review and meta-analysis. *Therapeutic Advances in Gastroenterology*.

[20] Tabrizi R., Ostadmohammadi V., Akbari M. (2019). The effects of probiotic supplementation on clinical symptom, weight loss, glycemic control, lipid and hormonal profiles, biomarkers of inflammation, and oxidative stress in women with polycystic ovary syndrome: a systematic review and meta-analysis of randomized controlled trials. *Probiotics and Antimicrobial Proteins*.

[21] Lavekar A. S., Raje D. V., Lavekar A. A. (2017). Role of probiotics in the treatment of nonalcoholic fatty liver disease: a meta-analysis. *Euroasian Journal of Hepato-Gastroenterology*.

[22] Sun J., Buys N. (2015). Effects of probiotics consumption on lowering lipids and CVD risk factors: a systematic review and meta-analysis of randomized controlled trials. *Annals of Medicine*.

[23] Moher D., Shamseer L., Clarke M. (2015). Preferred reporting items for systematic review and meta-analysis protocols (PRISMA-P) 2015 statement. *Systematic Reviews*.

[24] Shea B. J., Grimshaw J. M., Wells G. A. (2007). Development of AMSTAR: a measurement tool to assess the methodological quality of systematic reviews. *BMC Medical Research Methodology*.

[25] Gao X., Zhu Y., Wen Y., Liu G., Wan C. (2016). Efficacy of probiotics in non-alcoholic fatty liver disease in adult and children: a meta-analysis of randomized controlled trials. *Hepatology Research*.

[26] Ejtahed H.-S., Angoorani P., Soroush A.-R. (2019). Probiotics supplementation for the obesity management; A systematic review of animal studies and clinical trials. *Journal of Functional Foods*.

[27] Daniali M., Nikfar S., Abdollahi M. (2020). *A Brief Overview on the Use of Probiotics to Treat Overweight and Obese Patients*.

[28] Sotoudegan F., Daniali M., Hassani S., Nikfar S., Abdollahi M. (2019). Reappraisal of probiotics’ safety in human. *Food and Chemical Toxicology*.

